# The association between psychological characteristics and physical activity levels in people with knee osteoarthritis: a cross-sectional analysis

**DOI:** 10.1186/s12891-020-03305-2

**Published:** 2020-04-25

**Authors:** Daisuke Uritani, Jessica Kasza, Penny K. Campbell, Ben Metcalf, Thorlene Egerton

**Affiliations:** 1 Department of Physical Therapy, Faculty of Health Science, Kio University, 4-2-2, Umaminaka, Koryocho, Kitakatsuragigun, Nara, 6350832 Japan; 2 School of Public Health and Preventive Medicine, Monash University, 553 St Kilda Road, Melbourne, Victoria 3004 Australia; 3 Centre for Health, Exercise, and Sports Medicine, The University of Melbourne, 161 Barry Street, Melbourne, Victoria 3010 Australia

**Keywords:** Osteoarthritis, Knee, Pain, Pain catastrophizing, Fear of movement, Depression, Physical activity

## Abstract

**Background:**

The aim of this study was to examine the relationship between psychological characteristics and physical activity levels, measured as the average number of steps per day, in people with knee osteoarthritis (OA).

**Methods:**

This study analysed baseline data from a randomized controlled trial (Australian New Zealand Clinical Trials Registry reference: ACTRN12612000308897). A total of 167 adults aged over 50 years, with knee pain rated as four or more on an 11-point numeric rating scale, and knee OA diagnosed using American College of Rheumatology clinical criteria, were recruited from the community (62 men and 105 women, mean age, 62.2 ± 7.5 years). The average number of steps per day over seven consecutive days was measured using an accelerometer-based device. Psychological characteristics evaluated were: depressive symptoms (Depression Anxiety Stress Scale), self-efficacy (Arthritis Self-Efficacy Scale for pain and other symptoms), fear of movement (Brief Fear of Movement Scale for Osteoarthritis), and pain catastrophizing (Pain Catastrophizing Scale). The association between the average number of steps per day and psychological characteristics was analyzed using a multiple linear regression analysis, with the average number of steps per day as the dependent variable, adjusting for each psychological characteristic separately, and age, sex, body mass index, and pain entered as covariates.

**Results:**

There was evidence that the amount of physical activity was associated with fear of movement (coefficient [B]: − 117, 95% confidence interval [95%CI]: − 227 to − 8) and with pain catastrophizing (B: -44, 95%CI: − 86 to − 1). The association with self-efficacy was similar (B:117, 95%CI: − 12 to 246). However, the direction of the association with depressive symptoms was less clear (B: -59, 95%CI: − 138 to 19).

**Conclusions:**

The results of this study revealed that the relationship was such that lower fear of movement and lower pain catastrophizing may be associated with more steps per day. It may be hypothesized that fear of moving and pain catastrophizing lead to activity avoidance and that strategies to improve these disease-related psychological aspects may be useful in enhancing physical activity participation, although this hypothesis is highly speculative and needs further testing given the cross-sectional design of this study.

## Background

Knee osteoarthritis (OA) is a prevalent, costly chronic condition leading to pain and disability in older adults globally [1, 2]. People with knee OA are less physically active than the general population [3–5]. Increasing physical activity is important to manage the condition, as well as to address co-morbidities such as obesity, heart disease and diabetes that are commonly present in people with knee OA [6]. While clinical guidelines recommend that people with knee OA engage in physical activity [7, 8], changing physical activity behaviour is difficult. Understanding factors that are barriers to physical activity in this patient population may help direct the design and testing of interventions to increase physical activity.

Psychological impairments are common in people with knee OA and can include depressive symptoms [9], low self-efficacy for managing their OA symptoms [10], increased pain catastrophizing and increased fear of movement [11]. However, the relationship between these factors and physical activity in people with knee OA is unclear given limited and sometimes contradictory findings. Previous systematic reviews have reported no association between physical activity and depressive symptoms in people with knee OA [12, 13]. There is some evidence of a negative relationship with pain catastrophizing [14], and while self-efficacy in general is a consistent predictor of physical activity initiation and maintenance [15] and influences activity among older adults, self-efficacy related to symptom management has been less well studied. Fear of movement was discussed as a barrier to physical activity in a qualitative study of people with knee OA [16] but the association with actual activity has not been investigated. Most previous studies in OA assessed physical activity using questionnaires rather than objective methods such as accelerometry and did not necessarily control for other demographic factors that can influence the relationship such as education level and employment status.

The aim of this study therefore was to examine the relationship between selected psychological characteristics and mobility-related physical activity level, measured as the average number of steps per day, in people with knee OA. We hypothesized that greater self-efficacy for symptom management, less fear of movement and less pain catastrophizing would be associated, but depressive symptoms would not be associated with greater daily step count. Identifying psychological characteristics of those with more active lifestyles may be helpful in prescribing personalized programs to enhance physical activity in patients with knee OA who are inactive.

## Methods

This study analysed baseline data from a parallel, two-group randomized controlled trial that evaluated the effectiveness of adding telephone coaching to a physiotherapist-delivered physical activity intervention (Australian New Zealand Clinical Trials Registry reference: ACTRN12612000308897) [17].

### Participants

One hundred and sixty-eight adults were recruited from the community in Australia for a randomized controlled trial [17], through advertisements on the radio, social media and through a research volunteer database. One participant had missing data for the number of steps per day due to a technical problem with the activity monitoring device. Therefore, 167 of these participants (48 men, 37.2%) were included in the current study. Inclusion criteria were: aged over 50 years old; average knee pain rated as four or more on an 11-point numeric rating scale (NRS, range 0–10, higher = worse); knee OA diagnosed using American College of Rheumatology clinical criteria [18]; and sedentary or insufficiently physically active according to the Active Australia Survey (activity< 150 min or < 5 sessions in the past week) [19]. Exclusion criteria were: an inability to safely participate in moderate intensity exercise; undertaking regular lower extremity strengthening exercise or receiving non-pharmacological treatment for knee pain from a health professional more than once within the past six months; knee surgery or intra-articular corticosteroid injection within the past six months; history of joint replacement on study knee or on a surgical waiting list; systemic arthritic conditions; current or past (within four weeks) oral corticosteroid use; other condition affecting lower extremity function more than knee pain; and more than 21 on the depression subscale of the Depression Anxiety Stress Scale (DASS) [20] . Flow chart of participant recruitment was described in Additional file 1.

### Descriptive data

Height and weight were recorded by the physiotherapist at the clinical screening visit. Symptom duration, level of education and employment status were self-reported in the study questionnaire.

### Outcome measures

#### Physical activity

The average number of steps per day over seven consecutive days was measured using an accelerometer-based device (activPAL™ Professional). The activPAL™ was attached to the mid-anterior thigh with a re-usable gel PAL Stickie™, and further secured with a strip of Mefix (Mölnlycke Health Care AB, Sweden) medical grade adhesive bandage. Participants wore the activPAL™ continuously, except for when bathing and swimming [21]. The device was affixed to the participant by the research staff at the time they attended the research facility for clinical screening and baseline assessment. They were given a stamped addressed envelope to return the device by mail after 7 days. Participants were provided with written and verbal advice to remove the device if the skin became irritated and for bathing/swimming. They were provided with additional Mefix for replacing the device after bathing. All participants self-reported being able to follow the instructions and remove it only for short periods for bathing/swimming. Thus, all participants bar one had 7 days valid wear time for the calculation of their daily step count. The activPAL™ has been shown to be reliable and valid in older people [22], people who are overweight and sedentary [23], and people with chronic musculoskeletal pain [24].

#### Psychological questionnaires

Psychological characteristics were evaluated using self-report questionnaires completed at the time they attended the research facility for clinical screening and baseline assessment.

Depressive symptoms over the previous week were measured with the depression subscale of the DASS [25]. This 21-item short-form consists of seven questions for depression. Responses range from “0” (did not apply to me) to “3” (apply to me very much, or most of the time). Scores from the subscale are summed and multiplied by two to give a total score. The total score for this subscale ranges from 0 to 42 (higher scores indicate greater level of depressive symptoms).

Disease-specific self-efficacy for managing symptoms was measured using the Arthritis Self-Efficacy Scale (ASES) for pain (five items) and other symptoms (six items) [26]. Responses to each question range from one (very uncertain) to ten (very certain). Each subscale score was standardized with the total combined score for pain and other symptoms ranging from 2 to 20 (higher scores indicate higher self-efficacy).

Fear of movement was assessed using the Brief Fear of Movement Scale for Osteoarthritis (BFOMSO) [27]. It consists of six questions using a four-point scale from “strongly agree” to “strongly disagree” to assess fear of injury or re-injury due to movement. It ranges from six to 24 (higher scores indicate greater fear of movement).

Pain catastrophizing was assessed using Pain Catastrophizing Scale (PCS) [28]. It consists of 13 questions which measure tendencies to ruminate about pain, magnify pain, and feel helplessness about pain on scales from zero to four. The total score ranges from zero to 52 (higher scores indicate greater level of catastrophizing). Pain catastrophizing was only collected from 130 people out of 167 because of the high burden of the baseline questionnaire in the original study [17]. The PCS was one of several exploratory outcomes removed after 130 people had been enrolled in the original study [17].

### Statistical analysis

The association between amount of physical activity (average number of steps per day) and psychological characteristics were analyzed using multiple regression analysis, with the average number of steps per day as the dependent variable, adjusting for each psychological characteristic separately and age, sex, body mass index (BMI), pain, level of education, and employment status entered as covariates. Level of education was categorized “1; <3 years of high school”, “2; ≥3 years of high school”, and “3; Postsecondary”. Employment status was divided into “1; Currently employed” and “2; Not employed”. We assessed the assumptions of linear regression between the number of steps per day and other outcomes using standard residual plots. Statistical analyses were performed using SPSS software (version 22.0, SPSS Inc., Chicago, IL).

## Results

Participants’ characteristics, physical activity level and psychological measures are presented in Table 1. In summary, the cohort was predominantly women (63%), with mean ± standard deviation (SD) age of 62.2 ± 7.5 years and had high BMI (31.5 ± 7.1 kg/m^2^). The average number of steps per day ± SD was 7998 ± 2747 steps. Scatter plots between number of daily steps and psychological outcomes are depicted in Additional file 2.

**Table 1:** Participant characteristics and outcomes

Age, years	62.2 (7.5)
Symptom duration, years, no. (%)	
< 2	51 (31)
2–10	82 (49)
> 10	34 (20)
Height, cm	167.4 (9.5)
Weight, kg	88.3 (20.8)
Body Mass Index, kg/m^2^	31.5 (7.1)
Male, no (%)	62 (37)
Level of education, no. (%)	
< 3 years of high school	18 (11)
≥ 3 years of high school	63 (38)
Postsecondary	86 (52)
Employment status, no. (%)	
Currently employed	78 (47)
Not employed	89 (53)
Average number of steps per day	7998 (2747)
Pain, NRS (0–10)	5.7 (1.4)
Depression subscale of the DASS (0–42)	4.1 (4.7)
ASES for pain and other symptoms (2–20)	12.6 (2.8)
BFOMSO (6–24)	12.5 (3.2)
PCS (0–52)	14.8 (9.6)

All data are presented as mean (SD) unless otherwise stated, for the participants who provided accelerometry data (*n* = 167). *NRS* Numeric rating scale, *DASS* Depression Anxiety Stress scale, *ASES* Arthritis Self-Efficacy Scale, *BFOMSO* Brief Fear of Movement Scale for Osteoarthritis, *PCS* Pain Catastrophizing Scale

Multiple regression analysis revealed that for each unit increase in the BFOMSO, there was a reduction in the average number of steps per day of 117 steps (Table 2). Each unit increase in the PCS was associated with a reduction of 44 steps per day (Table 2). A one-unit increase in the ASES for pain and other symptoms was associated with a 117 average step per day increase (Table 2). A one unit increase in depression subscale of the DASS was associated with a decrease of 59 steps per day (Table 2). The results of the full regression models are depicted in Additional file 3.

**Table 2:** Association between number of steps/day and psychological outcomes

	B (95% CIs)	*p*-value	Adjusted R^2^
Depression subscale of the DASS	-59 (− 138–19)	0.138	0.34
ASES for pain and other symptoms	117 (−12–246)	0.075	0.34
BFOMSO	−117 (−227 – −8)	0.036	0.35
PCS	−44 (−86 – − 1)	0.044	0.35

*DASS* Depression Anxiety Stress Scale, *ASES* Arthritis Self-Efficacy Scale, *BFOMSO* Brief Fear of Movement Scale for Osteoarthritis, *PCS* Pain Catastrophizing Scale, B; regression coefficient

## Discussion

The results of this study indicate that there may be some association between average numbers of steps per day and psychological symptoms, with increased levels of fear of movement and pain catastrophizing associated with a reduction in the number of steps. Higher self-efficacy for symptom management may be associated with increased activity; however our results suggest depressive symptoms have a weak association with mobility-related activity.

Our results are consistent with previous research demonstrating greater fear of movement [11, 29–32] and greater pain catastrophizing [14, 29] are associated with less physical activity among several populations including patients with knee OA. Our quantitative findings are also supported by the fear-avoidance model [33], whereby pain catastrophizing and pain-related fear of movement are theorized to lead to avoidance of physical activity [33, 34]. Further, a qualitative study has demonstrated that fear of pain is a psychological barrier to engaging in physical activity in people with knee OA [16]. Our findings suggest that although there is evidence of associations between psychological characteristics and number of steps per day, these change in the number of steps per day given changes in psychological characteristics may be small. It is not unsurprising given the great number of variables that can be associated with an individual’s physical activity behaviour [35, 36]. At the same time, the large confidence intervals for the associations in this study is a limitation of the findings and suggests a larger sample is required.

In our study, fear of movement and pain catastrophizing were associated with activity after controlling for pain levels. This suggests that one’s personal attitude toward pain, rather than pain severity itself, is related to physical activity. However, other findings in the OA literature related to pain are conflicting. Some studies have demonstrated that higher pain is associated with lower physical activity in patients with knee OA [37, 38] while others have not [39]. Lazaridou et al. [14] similarly reported that the association between physical activity levels and pain intensity was moderated by pain catastrophizing. Interestingly, physical activity levels (measured via accelerometry) have not been found to increase following total joint replacement despite substantial improvements in pain [40].

Lower self-efficacy for managing OA pain and other symptoms may be related to lower physical activity levels as the standardized coefficient suggested a similar-sized relationship as fear of movement and pain catastrophizing (see Additional file 3), but the confidence interval around the standardized coefficient included zero indicating a lower level of confidence in the existence of a relationship (Table 2). Overall self-efficacy has previously been shown to be is a significant determinant of health behaviour [41]. Another study found that self-efficacy could be directly targeted by treatment to improve physical function for individuals with early knee OA [42]. Therefore, increasing self-efficacy for OA symptoms may also be a clinically important intervention goal for people with knee OA and warrants testing in a randomised controlled trial.

There is some conflict in the literature about the association between depressive symptoms and physical activity in the general population [43–45] and in people with knee OA [12, 13]. This may relate to differences in cohort characteristics and in study methodology including the measurement of depressive symptoms and defining and assessing physical activity. Our results showed no statistically significant association between depression levels and average daily steps. However, people who scored more than 21 on the depression subscale of the DASS [20] were excluded from the study itself, which constrained the spread of the data and may have influenced the results. The DASS scoring instructions indicate that the mean score for the participants in this study, i.e. 4.1, is in the normal range, so this may also be a reason why the observed associations included small values. Nonetheless, our finding of no statistically significant relationship concurs with that of two systematic reviews in people with knee OA [12, 13].

Although exploratory, our results suggest that fear of movement, pain catastrophizing and possibly self-efficacy for symptom management, are associated with physical activity behaviour, and therefore we speculate that efforts to increase walking behaviour in this patient population might be enhanced by strategies aimed at reducing fear of movement and pain catastrophizing, and improving self-efficacy for symptom management. There is evidence that specific pain neurophysiology education can reduce pain catastrophizing and increase knowledge about pain in people with chronic pain [46]. One of the primary aims of such education is to reconceptualise thinking about pain, away from the belief that “hurt” always equates to “physical harm”. Whether pain neurophysiology education subsequently increases physical activity levels has not yet been studied. Other psychological interventions may also be beneficial. Pain coping skills training, a form of cognitive behavioural therapy, has been investigated in people with knee OA. Bennell, et al. [47] found that pain coping skills training reduced pain catastrophizing but did not increase general physical activity levels unless it was combined with a structured exercise program. Further clinical trials are needed to investigate whether improvements in these psychological parameters mediate improvements in physical activity levels following targeted interventions.

Our study has several limitations. First, as this is a cross-sectional study, we cannot establish the temporal relationship between the psychological characteristics and physical activity levels. It is possible that being more physically active might lead to lower fear of movement and pain catastrophizing. The causal relationship needs to be investigated in randomized controlled trials. Second, we enrolled people classified as sedentary or insufficiently physically active based on the Active Australia Survey [19], a self-report instrument. This could have potentially constrained our ability to detect relationships in those with higher physical activity levels. However, based on our objective measure of physical activity, the average number of daily steps of the participants was indeed relatively high, around 8000. This would normally be deemed “somewhat active” [48]. A possible reason why participants had higher steps/day than expected is that wearing an accelerometer to measure daily steps might motivate participants to be more physically active. This limitation might confound true relationships between psychological status and physical activity levels. Third, participants were recruited for a clinical trial investigating an intervention that included physical activity. As such, results may not necessarily generalize to all people with knee OA as psychological characteristics may differ in those who volunteer for research of this nature.

The main strength of this study is that we described the association between the amount of objectively-recorded physical activity and psychological outcomes, and included multiple psychological constructs. Previous studies in OA assessed physical activity using self-report physical activity questionnaires, which lack demonstrated reliability and validity [49].

## Conclusions

The results of our study revealed that physical activity level, as indicated by the average number of steps per day, may be associated with fear of movement, pain catastrophizing and possibly self-efficacy for symptom management in people with knee OA, although the changes in the number of steps per day associated with changes in these variables may be small. It may be hypothesized that strategies to improve these psychological characteristics may be useful in enhancing physical activity behaviours, although this hypothesis needs testing given the cross-sectional design and exploratory nature of this study.

## Supplementary information

**Additional file 1.** Flow chart describing participant recruitment

**Additional file 2.** Scatter plots for number of steps per day and psychological characteristics

**Additional file 3.** Results of multiple regression analyses

## Data Availability

The datasets used and/or analysed during the current study are available from the corresponding author on reasonable request.

## Abbreviations

OA: Osteoarthritis
NRS: Numeric rating scale
DASS: Depression Anxiety Stress Scale
ASES: Arthritis Self-Efficacy Scale
BFOMSO: Brief Fear of Movement Scale for Osteoarthritis
PCS: Pain Catastrophizing Scale
BMI: Body mass index
