# Supplementary material for: The association between psychological characteristics and physical activity levels in people with knee osteoarthritis: a cross-sectional analysis
Source: BMC Musculoskelet Disord. 2020 Apr 25;21:269. doi: 10.1186/s12891-020-03305-2 (PMC7183118; doi:10.1186/s12891-020-03305-2)
Supplement: Supplementary file 1 — Additional file 1. Flow chart describing participant recruitment [file 12891_2020_3305_MOESM1_ESM.docx]

Assessed for eligibility by physical screening (n=205)

Not interested/lack of time (n=141)

Did not meet inclusion criteria (n=337)

Did not undergo physical screening (n=17)

Assessed eligibility by phone (n=700)

Did not meet physical screening inclusion criteria (n=3)

Passed screening but no longer met inclusion criteria (n=34)

Missing data for the amount of physical activity (n=1)

Included into the original study (n=168)

Included into the study (n=167)

Appendix 1. Flow chart describing participant recruitment
