# Supplementary material for: The association between psychological characteristics and physical activity levels in people with knee osteoarthritis: a cross-sectional analysis
Source: BMC Musculoskelet Disord. 2020 Apr 25;21:269. doi: 10.1186/s12891-020-03305-2 (PMC7183118; doi:10.1186/s12891-020-03305-2)
Supplement: Supplementary file 2 — Additional file 2. Scatter plots for number of steps per day and psychological characteristics [file 12891_2020_3305_MOESM2_ESM.docx]

a b

c d

Appendix 2. Scatter plots for number of steps per day and psychological characteristics

a. Depression subscale of the Depression Anxiety Stress Scale (DASS),

b. Pain and other symptoms subscales of the Arthritis Self-Efficacy Scale (ASES)

c. Brief Fear of Movement for Osteoarthritis (BFOMSO) d. Pain Catastrophizing Scale (PCS)
