# Supplementary material for: The association between psychological characteristics and physical activity levels in people with knee osteoarthritis: a cross-sectional analysis
Source: BMC Musculoskelet Disord. 2020 Apr 25;21:269. doi: 10.1186/s12891-020-03305-2 (PMC7183118; doi:10.1186/s12891-020-03305-2)
Supplement: Supplementary file 3 — Additional file 3. Results of multiple regression analyses [file 12891_2020_3305_MOESM3_ESM.docx]

Appendix 3. Results of multiple regression analyses

|  | B (95% Confidential Intervals) | β | p | Adjusted R^2^ |
| --- | --- | --- | --- | --- |
| **Depressive symptoms** |  |  |  | 0.34 |
| Intercept | 24764 (19978 – 29550) |  | <0.01 |  |
| Sex | -479 (-1222 – 265) | -0.08 | 0.205 |  |
| Age | -90 (-148 – -31) | -0.24 | 0.003 |  |
| BMI | -152 (-205 – -99) | -0.39 | <0.01 |  |
| Pain | -240 (-504 – 24) | -0.13 | 0.075 |  |
| Level of education | -1285 (-1842 – -728) | -0.32 | <0.01 |  |
| Employment status | -469 (-833 – -106) | -0.19 | 0.012 |  |
| DASS | -59 (-138 – 19) | -0.10 | 0.138 |  |
| **Self-Efficacy** |  |  |  | 0.34 |
| Intercept | 23507 (18643 – 28370) |  | <0.01 |  |
| Sex | -494 (-1236 – 248) | -0.09 | 0.190 |  |
| Age | -99 (-159 – -39) | -0.27 | 0.001 |  |
| BMI | -160 (-212 – -108) | -0.42 | <0.01 |  |
| Pain | -186 (-455 – 83) | -0.10 | 0.174 |  |
| Level of education | -1245 (-1794 – -696) | -0.31 | <0.01 |  |
| Employment status | -461 (-822 – -100) | -0.19 | 0.013 |  |
| ASES | 117 (-12 – 246) | 0.12 | 0.075 |  |
| **Fear of movement** |  |  |  | 0.35 |
| Intercept | 26408 (21330 – 31485) |  | <0.01 |  |
| Sex | -427 (-1162 – 309) | -0.08 | 0.253 |  |
| Age | -97 (-156 – -38) | -0.26 | 0.001 |  |
| BMI | -152 (-204 – -100) | -0.39 | <0.01 |  |
| Pain | -235 (-497 – 27) | -0.12 | 0.078 |  |
| Level of education | -1283 (-1833 – -734) | -0.32 | <0.01 |  |
| Employment status | -514 (-864 – -164) | -0.21 | 0.004 |  |
| BFOMSO | -117 (-227 – -8) | -0.14 | 0.036 |  |
| **Pain catastrophising** |  |  |  | 0.35 |
| Intercept | 24029 (18625 – 29434) |  | <0.01 |  |
| Sex | -381 (-1229 – 467) | -0.07 | 0.376 |  |
| Age | -74 (-140 – -9) | -0.20 | 0.027 |  |
| BMI | -147 (-207 – -87) | -0.37 | <0.01 |  |
| Pain | -252 (-554 – 50) | -0.13 | 0.102 |  |
| Level of education | -1201 (-1805 – -598) | -0.31 | <0.01 |  |
| Employment status | -527 (-930 – -124) | -0.22 | 0.011 |  |
| PCS | -44 (-86 – -1) | -0.15 | 0.044 |  |

DASS; Depression subscale of Depression Anxiety Stress Scale, ASES; Pain and other symptoms subscales of Arthritis Self-Efficacy Scale, BFOMSO; Brief Fear of Movement Scale for Osteoarthritis, PCS; Pain Catastrophizing Scale, B; regression coefficient, β; standardized partial regression coefficient
